# Stabilization of weak ferromagnetism by strong magnetic response to epitaxial strain in multiferroic BiFeO_3_

**DOI:** 10.1038/srep12969

**Published:** 2015-08-06

**Authors:** Hemant Dixit, Jun Hee Lee, Jaron T. Krogel, Satoshi Okamoto, Valentino R. Cooper

**Affiliations:** 1 Materials Science and Technology Division, Oak Ridge National Lab, Oak Ridge, TN 37830, USA; 2 Center for Nanophase Materials Science, Oak Ridge National Lab, Oak Ridge, TN 37830, USA

## Abstract

Multiferroic BiFeO_3_ exhibits excellent magnetoelectric coupling critical for magnetic information processing with minimal power consumption. However, the degenerate nature of the easy spin axis in the (111) plane presents roadblocks for real world applications. Here, we explore the stabilization and switchability of the weak ferromagnetic moments under applied epitaxial strain using a combination of first-principles calculations and group-theoretic analyses. We demonstrate that the antiferromagnetic moment vector can be stabilized along unique crystallographic directions ([110] and [–110]) under compressive and tensile strains. A direct coupling between the anisotropic antiferrodistortive rotations and the Dzyaloshinskii-Moria interactions drives the stabilization of the weak ferromagnetism. Furthermore, energetically competing *C*- and *G*-type magnetic orderings are observed at high compressive strains, suggesting that it may be possible to switch the weak ferromagnetism “on” and “off” under the application of strain. These findings emphasize the importance of strain and antiferrodistortive rotations as routes to enhancing induced weak ferromagnetism in multiferroic oxides.

The magnetoelectric effect (ME), i.e. controlling ferromagnetism using electric fields and vice versa, has been a focus of research in multiferroic materials due to their potential applications in technologies such as magnetic data storage, spintronics, logic and memory devices^1^,^2^,^3^,^4^. Unlike conventional magnetic storage devices where magnetic fields are used to read/write information, the ME utilizes electric fields for the read/write operations with virtually no power dissipation. This feature is promising not only for spintronics but also magnonics where the magnetic excitations (spin waves) are used to process information^3^. Although the ME is technologically attractive, the numbers of candidate materials remain limited. In this regard, bulk bismuth ferrite (BiFeO_3_) has been explored as a potential candidate. BiFeO_3_ exhibits coupling between ferroelectricity and ferromagnetism at room temperature^4^,^5^,^6^ and furthermore, the electric-field control of spin waves has been demonstrated^2^.

In the rhombohedral ground-state of BiFeO_3_, weak ferromagnetism (wFM) is induced from a slight canting of the collinear antiferromagnetic moments due to Dzyaloshinskii-Moria (DM) interactions^5^. Here, the strong coupling between atomic distortions and the DM interactions presents a route for tuning the wFM through changes in the lattice modes. It is well known that applied epitaxial strains can significantly affect the structure of the BiFeO_3_ perovskite unit cell resulting in polar cation shifts and/or a suppression or enhancement of the FeO_6_ octahedral tilt patterns; resulting in an array of material responses such as enhanced polarisation^7^ and high piezoresponse^8^ with implications for magnonic and spintronic responses^1^. Although the ferroelectric properties under applied epitaxial strain have been widely studied in both the tensile and compressive regimes, the evolution of the wFM under applied epitaxial strain remains relatively unexplored area^1^,^9^,^10^.

In this article, we systemically investigate the influence of epitaxial strain on the underlying mechanisms that link wFM with lattice distortions. For this purpose, we perform non-collinear spin-polarised density functional theory (DFT) calculations including spin-orbit couplings under applied compressive and tensile strains. Although we neglect the long-range spin-cycloid (62 nm period) phase that occurs in bulk BiFeO_3_^11^,^12^,^13^,^14^,^15^ and the possibility of thermal vibrations (or high temperature phonon-magnon couplings)^16^,^17^, our theoretical predictions should be relevant for thin films under epitaxial strain at low temperatures as this spin-cycloid is easily broken with a small amount of epitaxial strain (−1.7% ~ 0.5%)^1^. Here, we show that the wFM observed for the bulk rhombohedral structure persists for the tested strain values (±5%). Under moderate compressive strains (<−2%), the induced weak ferromagnetic moments due to spin canting remain quasi-degenerate in a plane perpendicular to the polarisation direction, but at higher compressive strains the antiferromagnetic vector (**L**) is stabilized along the [110] crystallographic direction. For tensile strains, on the other hand, **L** is switched by 90° and stabilized along the [–110] direction. We find that the stabilization of wFM is driven by the interplay between the Dzyaloshinskii-Moria (DM) interactions and the single ion anisotropy (SIA); with the DM interactions dominating under tensile strains and the SIA interactions having significant contributions under large compressive strains. Furthermore, we also observe that at high compressive strains (≥−7%), the *C-* and *G*-type magnetic orderings compete, energetically. Group-theoretic analyses indicate a loss of weak ferromagnetism for *C*-type magnetic ordering, which is also confirmed by DFT calculations. Thus, it may be possible to either switch on or switch off the weak ferromagnetism under external pressure. Our study offers useful insights for manipulating the distinct magnetic response of a material under epitaxial strain and has important implications towards utilizing the magnetoelectric effect in BiFeO_3_ thin films for future technology.

## Results

### Weak ferromagnetism in the ground state rhombohedral (*R3c*) structure

**Figure 1:**
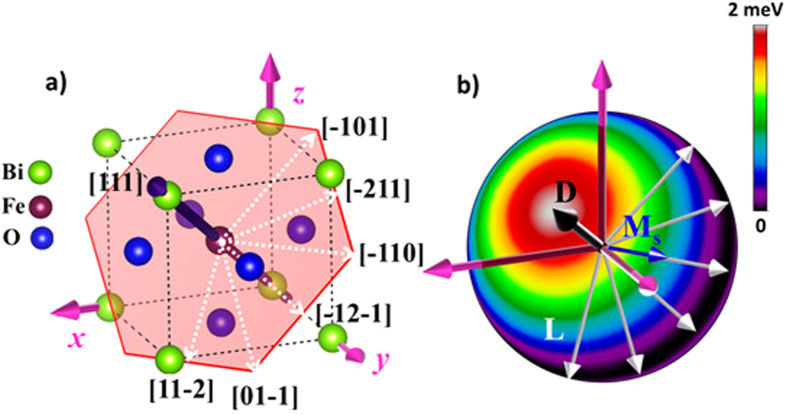
(**a**) Schematic of the BiFeO_3_ crystal structure showing the polarisation direction and the plane (red hexagon) perpendicular to the [111] polarisation direction (**b**) Calculated magnetic energy landscape the bulk rhombohedral phase. The dark band in this plot depicts all possible orientations of the easy spin axis which are found to be degenerate in the (111) plane perpendicular to the [111] direction. Consequently, the spontaneous weak ferromagnetism (**M**_**s**_) is also degenerate in the (111) plane in such a way that **D**, **L** and **M**_**s**_ forms a right handed system.

Non-collinear DFT calculations within the LDA + *U* formalism including spin-orbit coupling find that the magnetic ground state of the rhombohedral (*R3c*) structure exhibits *G*-type anti-ferromagnetic ordering. Furthermore, these collinear magnetic moments are canted due to the DM interactions and result in an induced wFM. In agreement with previous first principles calculations^18^, we find that the easy spin axis (i.e. the direction of the induced wFM) is degenerate and lies in a plane perpendicular to the polarisation ([111]) direction. Thus the weak ferromagnetic moments may lie along any one of six possible crystallographic directions ([10–1], [–211], [–110], [–121], [01–1] and [11–2]) which are perpendicular to the polarisation vector^19^, (see Fig. 1a). Here, we make use of spherical polar coordinates (*m*, *θ and φ*) to calculate the magnetic energy landscape along all possible crystallographic directions. For this purpose, *m* is taken as the magnetic moment on an Fe atom, *θ* is defined as the angle between the initial collinear arrangement of the magnetic moments and the *z*-axis (*θ* ∈ [0; 180]) and *φ* is the angle between the magnetic moment and the *x*-axis (*φ* ∈ [0; 360]) along which the magnetic moments are constrained. Both *θ* and *φ* are varied in steps of 15°, resulting in a total of 288 non-collinear calculations at each strain value. These calculations were performed in a high-throughput fashion using the Nexus workflow automation system^20^. As depicted in Fig. 1b), we observe that the maximum energy configuration (the bright red and white region) corresponds to *θ* = 54.73 and *φ* = 45°, which is precisely the direction of the polarisation vector along the [111] direction. The minimum energy configurations (dark black region), on the other hand, show that the antiferromagnetic vector (**L**) is degenerate in a plane that is perpendicular to the polarisation vector. Thus, as a consequence the easy spin axis corresponding to the induced weak ferromagnetism is also degenerate in the (111) plane. An energy difference of ~2 meV was observed between magnetic moments aligned parallel and perpendicular to the polarisation vector.

In addition, our analysis of the magnetic ground state shows that the magnetic moments are canted by ~1° away from their initial collinear arrangement, resulting in a small but measurable spontaneous net magnetisation (**M**_**s**_) of 0.033 μB/Fe in good agreement with earlier reports^10^,^18^. Moreover, this value of magnetization also agrees quite well with various thin film measurements^21^,^22^,^23^, justifying the choice of the Hubbard parameter (*U*_*eff*_ = 2 eV) in the calculations. To elucidate the observed degeneracy of the easy spin axis in the plane perpendicular to the polarisation direction, we explicitly calculated the DM interactions for the rhombohedral structure (refer to the Methods section for details). For the *R3c* symmetry, the DM vector points along the [111] direction with isotropic local components i.e. |**D**_1_| = |**D**_2_| = |**D**_3_|. Consequently, wFM is degenerate in a plane perpendicular to the DM vector since **D**, **L** and **M**_**s**_ form a right-handed system. The calculated strength of the DM interaction is 304 μeV. (N.B. although this value is overestimated compared to the experimental value of 162 μeV^24^ due to the chosen Hubbard parameter (*U*_*eff*_ = 2 eV); we expect the predicted trends to remain valid. We have verified this with calculations employing a higher *U*_*eff*_ (=5 eV) value which reduces the strength of the DM interaction to 182 μeV, in good agreement with experiment. However, higher values of *U*_*eff*_ also lead to larger deviations in lattice parameter and antiferrodistortive angles, hence, in this study we use *U*_*eff*_ = 2 eV which gives a more reliable description of the structure).

### Coupling between the antiferrodistortive rotations and ferroelectric polarisation

**Figure 2:**
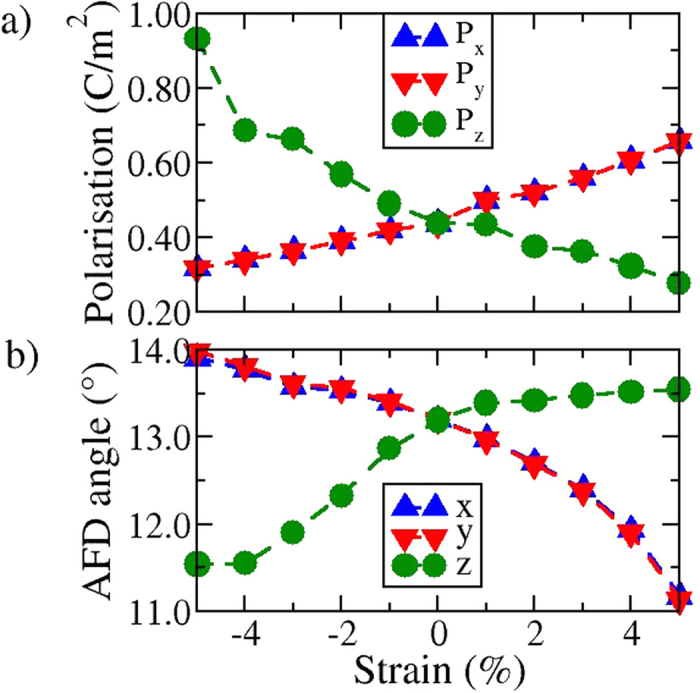
Variation in the in-plane (*x*-*y*) and out-plane (*z*) components of the (**a**) polarisation (**P**) and (**b**) antiferrodistortive (AFD) rotations under applied epitaxial strain.

We now discuss the changes in the electric polarisation direction and the AFD rotations with respect to applied epitaxial strain. Under applied compressive strains, the polarisation vector rotates along the (–110) plane towards the [001] direction (decreasing *θ*, while *φ* remains constant), away from the *R3c* [111]. On the other hand, for tensile strains the polarisation vector rotates towards the [110] direction along the (–110) plane. The rotation of the polarisation vector is strongly correlated with the FeO_6_ octahedral rotation patterns. Figure 2 summarises the changes in the *x*, *y* and *z*-components of the polarisation vector and the antiferrodistortive (AFD) rotations as a function of applied epitaxial strain. For the rhombohedral ground state, the calculated out-of-phase rotation of the FeO_6_ octahedra about the [111] direction is 13.2°, which is in good agreement with the experimentally measured value of 12.8°^25^. For compressive strains, we observe that the in-plane (x and y) components of the AFD rotations increase from 13.2° to 14°, while the *z*-component, rapidly decreases from 13.2° to 11.5°. For tensile strains, the *x-* and *y*-components of the AFD rotations rapidly decrease from 13.2° to 11° while the *z*-component remains nearly constant.

### Stabilization of weak ferromagnetic moments under applied epitaxial strain

**Figure 3:**
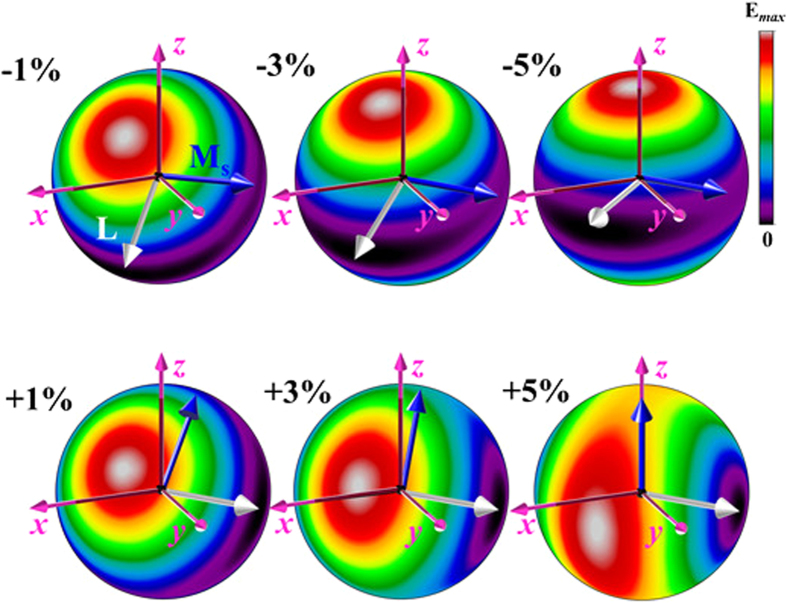
Calculated magnetic energy landscape for compressive (–) and tensile (+) strains of ±1, 3 and 5%. Bright (red) and dark (black) region correspond to hard and easy spin axes (also marked using dashed white arrows), respectively. Under both the compressive and tensile strains the antiferromagnetic vector (**L**) is stabilized along [110] and [–110] directions, respectively. Consequently, the induced weak ferromagnetism (**M**_**s**_) is also stabilized along the [–110] and [001] directions under the compressive and tensile strains, respectively.

Next, we discuss the evolution of the induced weak ferromagnetism under applied epitaxial strain. Figure 3 shows the calculated magnetic energy landscapes under applied compressive and tensile strains. We observe that the wFM observed in the case of the rhombohedral ground state also persists for the tested strain values (±5%). In the tensile regime (the lower panel of Fig. 3), we observe that the degeneracy of the induced weak ferromagnetic moments is quickly lifted resulting in a preferred orientation along specific crystallographic directions. A careful analysis of the wFM shows that both the easy and hard spin axes now lie in the *x*-*y* plane and **L** is stabilized along the [–110] direction. The spontaneous magnetisation is found to linearly decrease from 0.033μB for the *R3c* structure to 0.026μB for the highest strained (+5%) phase.

For the compressive regime, the magnetic energy landscape depicts a hard spin axis that closely follows the rotating polarisation vector with increasing compressive strain; again, due to the right hand rule **L** is always perpendicular to this direction. Furthermore, the weak ferromagnetic moments are quasi-degenerate in a plane that is perpendicular to the polarisation vector for moderate compressive strains (≤2%). For higher strain values, however, the degeneracy disappears and **L** is stabilized along the [110] crystallographic direction. Similar to the tensile strained systems, the spontaneous magnetisation decreases almost linearly from 0.033μB for the *R3c* structure to 0.028μB for the highest strained (−5% strain) phase.

### Colossal response in Dzyaloshinskii-Moria interactions and single ion anisotropy

**Figure 4:**
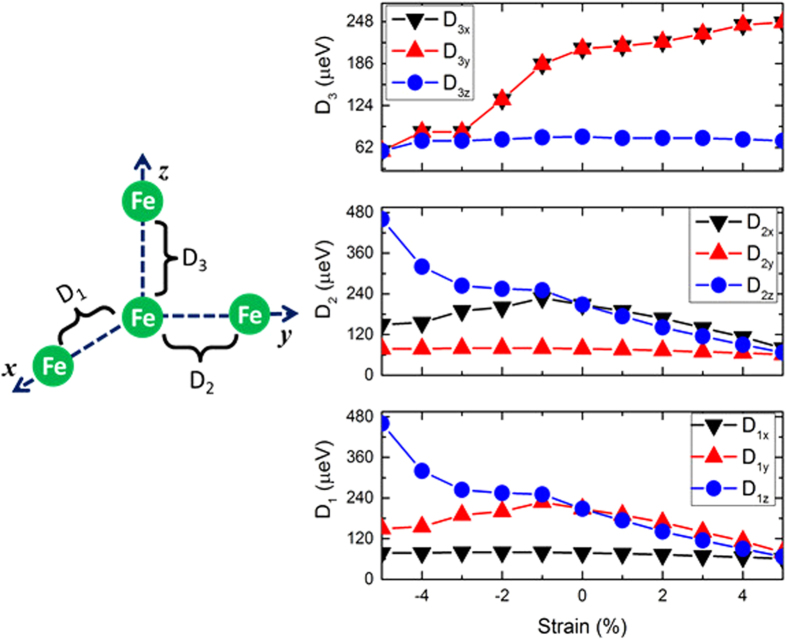
Strain dependence of the in-plane (*x*-*y*) and out-plane (*z*) components of the local DM interactions.

**Figure 5:**
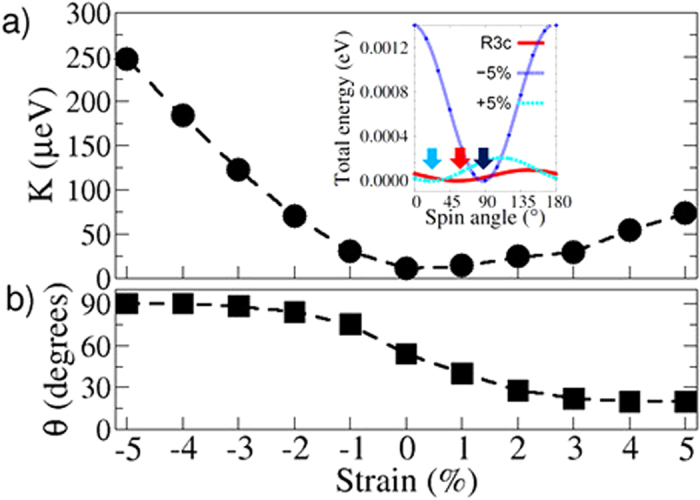
Calculated single ion anisotropy vector (*K***n**) as a function of applied epitaxial strain. (**a**) The magnitude and (**b**) The direction of the easy spin axis with respect to the *z*-axis. The inset shows the relative sinusoidal variations of the single ion anisotropy. The energy minimum (indicated with red arrow) for the bulk ground state corresponds to 54° i.e. along the [111] direction. The applied epitaxial strain shifts the energy minimum (indicated using black and cyan arrows) toward the *z*-axis and the *x*-*y* plane for compressive and tensile strains, respectively.

To analyse the spin polarisation along the unique [110] and [–110] directions, we calculated the DM interactions and SIA for each strained phase. It is important to recall that the spin canting is induced by the AFD rotations and hence, the wFM and by extension the DM interactions may be directly linked to the amplitude of these distortions. The variations in the components of the local DM vector (**D**_{1,2,3}_) as a function of applied epitaxial stain are shown in Fig. 4. For the rhombohedral structure, we have isotropic DM interactions with |**D**_1_| = |**D**_2_| = |**D**_3_| = 304 μeV. On the other hand, the calculated SIA (refer to Fig. 5) is merely 11 μeV (which compares well with the experimentally measured value of 3.5 μeV^26^); significantly lower than the DM interactions and thus does not compete with the DM interactions. It is interesting to note that the easy spin axis (**n**) actually points exactly along the [111] direction. However, since the energy gain from the isotropic DM interactions is dominant, net spins are polarised along a direction perpendicular to [111]. Furthermore, the rotational symmetry promotes the degeneracy of the easy spin axis in the (111) plane. Hence, as a result of the isotropic DM interactions pointing along the [111] direction both **L**, the induced weak ferromagnetic moments are degenerate in a plane perpendicular to the polarisation direction.

For the tensile region, we observe that the in-plane components of the DM vector are reduced as a consequence of broken rotational symmetry and reduced *x*-*y* components of the AFD rotations. The DM vectors move away from the *z*-axis along the (–110) plane. For the highest strained phase (5%), the components of the local DM vector are anisotropic and the DM vector actually points along the [11ε] (ε ≪ 1) direction. The SIA moderately increases from 11 μeV corresponding to the *R3c* structure to 73 μeV for the highest strained phase and the SIA vector (**n**) makes an angle of ~20° with respect to the *z*-axis as shown in Fig. 5b. The SIA, however, is still much smaller than the predicted DM interactions. Thus, even though the SIA is pointed along the *z*-axis, the stabilization of the **L** vector corresponding to the magnetic ground state is along the [–110] crystallographic direction due to the dominant DM interactions.

Under compressive strain, on the other hand, we observe that the in-plane (**D**_**1**_, **D**_**2**_) components of the DM vector are enhanced as a consequence of the broken rotational symmetry. The DM vector closely follows the polarisation direction and rotates towards the *z*-axis in the (–110) plane. The weak ferromagnetism, **M**_s_, is related to the sign of **D** in such a way that the three vectors - **D**, **S**_1_ and **S**_2_ form a right handed system^27^. As the DM vector rotates towards the *z*-axis, the antiferromagnetic vector, **L,** (which is perpendicular to the DM vector) rotates towards the *x*-*y* plane. In this process, only a few crystallographic directions that are commensurate with the anisotropic DM vector remain degenerate in the magnetic energy landscape. Additionally, we observe that the SIA is significantly enhanced from 11 μeV (for the rhombohedral ground state) to 247 μeV for the highest compressively strained phase and now competes with the DM interactions (|**D**_1_| = |**D**_2_| = 364 μeV |**D**_3_| = 98 μeV). Moreover, the SIA vector (**n**) lies in the *x*-*y* plane. Thus, because of the strongly enhanced SIA, **L** is stabilized along the [110] direction for the highest strained phase.

The atomistic origin of the enhancement in the DM interactions can be attributed to the changes in the AFD rotations. We observe that the strength of the DM interactions is proportional to the amplitude of the AFD rotations, under both compressive and tensile strains, which exhibit a direct coupling between the DM interactions and the lattice modes. The changes in the SIA, on the other hand, are related to the Fe-O hybridization. The spin-orbit coupling induced energy correction is proportional to the extent of *p*-*d* hybridization between the O-*p* and Fe-*d* states. In the case of compressive strains where the in-plane Fe-Fe separation is reduced and consequently the Fe-O hybridization increases, the SIA is enhanced in the *xy* plane. On the other hand, tensile strains reduce the out-of-plane Fe-Fe separation and enhance the out of plane Fe-O hybridization thereby rotating the SIA towards the *z*-axis away from the [111] direction.

### Symmetry-driven magnetic switch at high compressive strain

**Figure 6:**
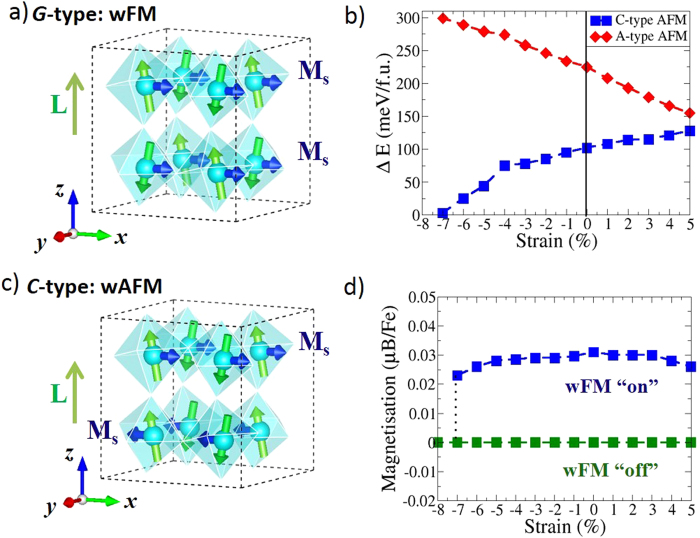
Schematic of the induced weak ferromagnetism (shown with blue arrows) due to spin canting for (**a**) *G*- and (**b**) *C*-type magnetic ordering. For *C*-type magnetic ordering, by symmetry, the DM interactions in adjacent layers oppose each other giving rise to wAFM. (**c**) The total energy differences as a function of applied strain for *A* and *C*-type magnetic ordering relative to the *G*-type ground state and (**d**) the induced magnetisation for *C* and *G*-type magnetic ordering. The crystal structures are generated using VESTA^34^.

So far, we have discussed the evolution of the weak ferromagnetism with *G*-type magnetic ordering. Here, we examine the possibility of magnetic switching under applied epitaxial strain. Figure 6b shows the total energy difference along with the induced wFM moments for the *A*- and *C*-type magnetic orderings relative to the ground state *G*-type order. For all strain values studied, the *G*-type magnetic ordering is the ground state magnetic configuration. The non-magnetic ordering is typically ~1.0–1.4 eV higher in energy compared to the *G*-type magnetic ordering indicating that the antiferromagnetic order is robust at room temperature (see supplementary information Table S1 for details). Furthermore, the *A*-type magnetic ordering is always higher in energy compared to the *C*- and *G*-type magnetic orderings. Under compressive strains, the energy difference between the *C*- and *G*-type magnetic orderings decreases rapidly with increasing strain and at high strain values (≥7%) the energy difference is nullified making these two types of magnetic orderings indistinguishable. Such indistinguishability between the energetically competing *C-* and *G*-type magnetic orderings has been observed in experiments for the tetragonal like (*T’*) monoclinic phase at ~4.5% compressive strain^28^. Most interestingly, a symmetry analysis for the *C*-type magnetic ordering shows that the DM interactions of neighbouring planes (refer to the supplementary information Table S2) result in weak antiferromagnetism (wAFM). The energetic similarity of the *C*- and *G*-type antiferromagnetic ordering at large compressive strains and the transition from net wFM for the *G*-type ordering to net wAFM for *C*-type ordering, suggest that it may be possible to switch the wFM “on” or “off” through the application of external pressure or modulation of lattice modes in these materials.

## Discussion

In summary, we have studied the effects of applied biaxial compressive and tensile strains on the evolution of the net weak ferromagnetism in BiFeO_3_. The magnetic energy landscapes, constructed from non-collinear spin polarised DFT calculations, indicate that induced weak ferromagnetic moments are always aligned perpendicular to the polarisation vector and persist for strains up to ±5%. Furthermore, the spontaneous net magnetisation is only marginally reduced for large strains compared to the bulk. For the rhombohedral structure the weak ferromagnetic moments are degenerate (in plane perpendicular to the polarisation direction) due to the isotropic nature of the DM interactions and the rotational symmetry. Such degeneracy, however, disappears for the strained phases and the antiferromagnetic vector is stabilized along [110] and [–110] crystallographic directions for compressive and tensile strains, respectively. The stabilization of the magnetic moments is correlated with the reduction of AFD rotations along specific crystallographic axes (*z*-axis for compressive strains and *x*-*y* for tensile strains); resulting in anisotropic DM interactions. The strength of the DM interactions is proportional to the AFD rotations, which opens a possibility for enhancing the induced wFM through modulation of the lattice modes. Furthermore, at high compressive strains both the *C*- and *G*-type magnetic orderings compete. Group theoretic analyses indicate the loss of weak ferromagnetism for the *C*-type magnetic order. Thus, the wFM can be switched “on” and “off” depending on the type of magnetic ordering. These results have profound implications for using multiferroic oxide films in spintronics devices where it is possible to use epitaxial strain or external pressure to control the spin polarisation along certain crystallographic directions.

### Method and Computational Details

All calculations were performed using density functional theory (DFT) with the local spin density approximation (LSDA) employing the projector-augmented-plane-wave (PAW) method^29^, as implemented in the Vienna Ab initio Simulation Package (VASP 5.2)^30^,^31^. The PAW potentials used explicitly treat 15 valence electrons for Bi (5d^10^ 6s^2^ 6p^3^), 14 for Fe (3p^6^ 3d^6^ 4s^2^) and 6 for oxygen (2s^2^ 2p^4^). A cut-off energy of 520 eV was used to terminate the planewave expansion. We considered a 2 × 2 × 2 supercell containing 40 atoms which can accommodate all the possible antiferrodistortive rotations of the FeO_6_ octahedra. Structural optimizations were achieved by allowing the atoms in the unit cell to relax until the Hellmann-Feynman forces on each atomic site were below 5 meV/Ǻ, while simultaneously achieving a total energy convergence of 1e^−6^ eV. This convergence was obtained with a 4 × 4 × 4 Monkhorst-Pack special *k*-point grid. To correct for the metallic behavior observed in the LDA band structure we have applied the Hubbard parameter, *U*_*eff*_ = 2 eV^32^, on Fe-*d* states in our calculations using the rotationally invariant scheme of Dudarev *et al*.^33^ in which the on-site Coulomb interaction (U) and parameter J are combined into a single parameter *U*_eff_ (=*U – J*).

The DM interaction has the following form:


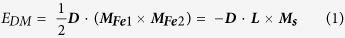


Here, **D** is the coupling vector for the spin-orbit interaction, **L** is the antiferromagnetic vector defined as **L** = **M**_Fe1_ − **M**_Fe2_ and **M**_**s**_ is the resulting magnetisation due to the canted moments (**M**_**s**_ = **M**_Fe1_ + **M**_Fe2_). These three vectors (**D**, **L** and **M**_**s**_) form a right-handed system and for a fixed orientation of **D** and **L**, only one canting direction lowers the energy relative to the collinear state. We calculate the DM vectors (**D**_{1,2,3}_) and SIA using the method proposed by C. Weingert *et al*.^27^. For this purpose, we used 2 × 2 × 2 supercells containing 8 formula units. Artificial calculations are performed by selecting two Fe atoms along either *a*, *b* or *c*-axis directions and replacing the remaining Fe atoms in the supercell with non-magnetic Al ions. The DM vectors can then be extracted using a perpendicular arrangement of spins with:


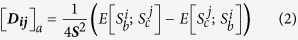


The three local planar components **D**_1_, **D**_2_ and **D**_3_ lie in the *x*-*y*, *y*-*z* and *x*-*z* planes, respectively.

The SIA is calculated by replacing all except one of the Fe atoms with non-magnetic Al atoms and later performing constrained calculations along all possible directions to calculate the SIA vector **n**. The magnitude of SIA, *K,* was obtained by fitting the following expression:


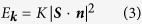


with **n** = 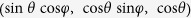 in the spherical coordinate. In our simulations, 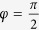 is always realized.

## Additional Information

**How to cite this article**: Dixit, H. *et al.* Stabilization of weak ferromagnetism by strong magnetic response to epitaxial strain in multiferroic BiFeO_3_. *Sci. Rep.* **5**, 12969; doi: 10.1038/srep12969 (2015).

## Supplementary Material

Supplementary Information
