# Supplementary material for: Stabilization of weak ferromagnetism by strong magnetic response to epitaxial strain in multiferroic BiFeO3
Source: Sci Rep. 2015 Aug 6;5:12969. doi: 10.1038/srep12969 (PMC4526888; doi:10.1038/srep12969)
Supplement: Supplementary Information [file srep12969-s1.doc]

**Supplementary information::**

**Stabilization of weak ferromagnetism by strong magnetic response to epitaxial strain in multiferroicBiFeO_3_**

*Hemant Dixit^1,*^, Jun Hee Lee^1,*^, Jaron T. Krogel^1,2^, Satoshi Okamoto^1^ and Valentino R. Cooper^1,*^*

1. *Materials Science and Technology Division, Oak Ridge National Lab, Oak Ridge, TN*

*37830, USA*

1. *Center for NanophaseMaterials Science, Oak Ridge National Lab, Oak Ridge, TN*

*37830, USA*

*Email: [dixithm@ornl.gov](mailto:dixithm@ornl.gov),[leej@ornl.gov](mailto:leej@ornl.gov), [coopervr@ornl.gov](mailto:coopervr@ornl.gov)

**Table S1**. Total-energy difference (in meV/f.u) between different types of magnetic orderings and the ground-state *G*-type antiferromagnetic ordering under applied epitaxial strain.

| Strain | A-type | C-type | Ferromagnetic | Non-magnetic |
| --- | --- | --- | --- | --- |
| -5 | 155 | 128 | 380 | 1452 |
| -4 | 166 | 121 | 405 | 1434 |
| -3 | 179 | 114 | 391 | 1395 |
| -2 | 193 | 115 | 392 | 1292 |
| -1 | 210 | 108 | 394 | 1238 |
| 0 | 222 | 101 | 331 | 1101 |
| 1 | 234 | 96 | 373 | 1126 |
| 2 | 246 | 85 | 365 | 1131 |
| 3 | 258 | 78 | 348 | 1139 |
| 4 | 274 | 75 | 342 | 1180 |
| 5 | 279 | 44 | 344 | 1247 |

The wFM ordering is then a consequence of the cross product of the antiferromagnetic (-), ferromagnetic (+) order and the in-phase (-), out-of-phase (+) octahedral rotation patterns along the crystallographic *a*,*b* and *c* directions. For *G*-type magnetic ordering the wFM moments are coupled in both the in-plane and out-plane directions, whereas for *C*-type magnetic ordering they are ferromagnetically coupled in-plane but are antiferromagnetically coupled out-of-plane resulting in wAFM. The **Table S2** below summarizes the relation between the magnetic order and AFD rotations leading to wFM (+) or wAFM (-) order along the crystallographic *a*,*b* and *c* directions.

**Table S2**. Relation between the magnetic order (ferromagnetic:’+’, antiferromagnetic:’-‘) and AFD rotations (in-phase:’+’, out-phase:’-‘) leading to wFM (+) or wAFM (-) order along the crystallographic *a*,*b* and *c* directions.

|  | **a** | **b** | **C** |
| --- | --- | --- | --- |
| ***G*-type (*G*)** | - | - | - |
| ***C*-type (*C*)** | - | - | + |
| **AFD rotations (R)** | - | - | - |
| ***G*×R** | + | + | + |
| ***C*×R** | + | + | - |
